# High-flow nasal oxygen in acute hypoxemic respiratory failure: A narrative review of the evidence before and after the COVID-19 pandemic

**DOI:** 10.3389/fmed.2022.1068327

**Published:** 2022-11-25

**Authors:** Léon Genecand, Thomas Agoritsas, Charlotte Ehrensperger, Aileen Kharat, Christophe Marti

**Affiliations:** ^1^Division of General Internal Medicine, Geneva University Hospitals, Geneva, Switzerland; ^2^Faculty of Medicine, University of Geneva, Geneva, Switzerland; ^3^Department of Health Research Methods, Evidence and Impact, McMaster University, Hamilton, ON, Canada; ^4^Division of Pulmonary Medicine, Geneva University Hospitals, Geneva, Switzerland

**Keywords:** high flow nasal oxygen, non-invasive ventilation, continuous positive air pressure, bi-level airway pressure, acute hypoxemic respiratory failure, COVID-19

## Abstract

High-flow nasal oxygen (HFNO) is a type of non-invasive advanced respiratory support that allows the delivery of high-flow and humidified air through a nasal cannula. It can deliver a higher inspired oxygen fraction than conventional oxygen therapy (COT), improves secretion clearance, has a small positive end-expiratory pressure, and exhibits a washout effect on the upper air space that diminishes dead space ventilation. HFNO has been shown to reduce the work of breathing in acute hypoxemic respiratory failure (AHRF) and has become an interesting option for non-invasive respiratory support. Evidence published before the COVID-19 pandemic suggested a possible reduction of the need for invasive mechanical ventilation compared to COT. The COVID-19 pandemic has resulted in a substantial increase in AHRF worldwide, overwhelming both acute and intensive care unit capacity in most countries. This triggered new trials, adding to the body of evidence on HFNO in AHRF and its possible benefits compared to COT or non-invasive ventilation. We have summarized and discussed this recent evidence to inform the best supportive strategy in AHRF both related and unrelated to COVID-19.

## Introduction

Acute hypoxemic respiratory failure (AHRF) is defined by the presence of hypoxemia without significant hypercapnia. AHRF is usually caused by inadequate ventilation-to-perfusion ratios resulting in poorly oxygenated blood being allowed into the systemic circulation. It can be caused by various conditions including bacterial or viral pneumonia, cardiogenic pulmonary edema, atelectasis, respiratory disease exacerbations—e.g., from chronic obstructive pulmonary disease (COPD)—pleural effusion, or a combination of either of these conditions. AHRF may occur spontaneously or in post-operative, post-extubation, or trauma patients. These different settings involve distinct underlying mechanisms leading to AHRF, present a different prognosis, and are thus usually discussed separately in the clinical practice guidelines on HFNO and non-invasive ventilation (NIV) (1–4). In this present review, we will focus solely on *de novo* AHRF. We will use the term “AHRF” when not referring to a specific etiology and the term “*de novo* AHRF” when referring to AHRF occurring without underlying pulmonary disease and excluding cardiogenic pulmonary edema.

Acute respiratory distress syndrome (ARDS) is the most severe form of AHRF. ARDS is defined by the Berlin criteria as follows: new-onset, bilateral pulmonary opacities consistent with pulmonary edema with a PaO_2_/FiO_2_ of ≤ 300 mmHg under a minimal positive end-expiratory pressure (PEEP) of ≥5 cmH_2_O (5). One must first exclude a cardiac origin from pulmonary edema. The severity is determined by the PaO_2_/FiO_2_ ratio. ARDS carries a poor prognosis with a mortality ranging from 27% for mild ARDS to 45% for severe ARDS (5). The treatment is mainly supportive, focusing on the patient's oxygenation. Historically, conventional oxygen therapy (COT) has been used—nasal cannulas, facemasks, non-rebreather facemasks, or Venturi masks—in association with low-tidal volume invasive mechanical ventilation (IMV) for the most severe patients. However, IMV is associated with several complications, such as ventilator-associated pneumonia, delirium, or ICU-associated polyneuropathy, and is resource-demanding (1). Therefore, non-invasive respiratory support strategies, including continuous positive airway pressure (CPAP), bi-level positive airway pressure (BiPAP), and high-flow nasal oxygen (HFNO), have been explored as possible alternatives to IMV (1–4).

The COVID-19 pandemic resulted in a substantial increase in both AHRF and ARDS overwhelming both acute and intensive care unit capacity, urging the exploration of alternative strategies to avoid the need for IMV and preserve ICU capacity. In this context, HFNO and NIV (CPAP or BiPAP) have been proposed as alternative strategies to COT to avoid IMV. Several observational and randomized controlled trials assessed their impact on mortality and the need for IMV (6–15).

Before reviewing this recent clinical evidence, we will first review the physiological basis and characteristics of these different types of non-invasive respiratory support and then discuss conflicting recommendations in recent international guidelines (3, 4).

## Physiological aspects of non-invasive respiratory support types

The main therapeutic objective in *de novo* AHRF is to provide sufficient oxygenation and to prevent respiratory exhaustion, which will then require IMV. Various techniques of administration of supplemental oxygen and/or positive end-expiratory pressure serve this purpose. Additional inspiratory pressure may or may not be applied.

### COT

COT is the most widely used technique to provide supplemental oxygen. COT is easy to use and delivers a flow of dry oxygen through a nasal cannula or facial interface to increase the inspired fraction of oxygen (FiO_2_). Indeed, the actual FiO_2_ depends on the flow of oxygen, the interface (nasal cannula, facemask, non-rebreathing facemask, or Venturi), and the inspiratory flow of the patient. In patients with *de novo* AHRF, the peak inspiratory flow is high with reported means of 30–40 L/min and up to 120 L/min (16), which greatly exceeds the flow delivered by most COT devices (up to 4 L/min with a nasal cannula, up to 8 L with a facial mask, and up to 10–15 L/min with a non-rebreathing mask). Consequently, FiO_2_ delivered by COT is imprecise, often overestimated (2), and cannot exceed 70% (17). Higher flows are not used with COT due to poor tolerance in the absence of humidification or air warming. Moreover, COT only increases the FiO_2_ but does not improve the recruitment or ventilation/perfusion ratio nor reduce the work of breathing.

### HFNO

HFNO can deliver warm and humidified air with a flow of up to 70 L/min (18). The delivery of high flow allows a fraction of inspired oxygen (FiO_2_) up to 100%, a washout effect of the upper airway, diminishing the dead space ventilation, and a positive expiratory effect pressure (PEEP) from 2 to 5 cmH_2_O depending mainly on the intensity of the flow, the tightness of the nasal cannula seal, and the patient's degree of mouth opening (19). The inflow of warm and humidified air improves device tolerance and may help muco-ciliary clearance (2). In AHRF, HFNO may reduce the work of breathing through various mechanisms including the inflow of warm and humidified air, the washout effect, and the effect on lung volume and compliance through the PEEP effect (20).

### NIV

In the published literature, the terminology of NIV often encompasses both continuous positive airway pressure (CPAP) and bi-level positive airway pressure (BiPAP) (1). We use the same terminology in the present review, even though CPAP does not have an effect on ventilation *per se* and some authors suggest that the use of the term NIV should be restricted to BiPAP (21). In addition, CPAP and BiPAP have distinct clinical indications (1).

CPAP consists of the administration of continuous airway pressure independent of the phase of the respiratory cycle. This pressure will thus also be applied at the end of expirium and be equivalent to the positive end-expiratory pressure (PEEP). The use of CPAP reduces the right ventricle preload and decreases the left ventricle afterload. CPAP increases the functional residual capacity improving alveolar recruitment, decreasing atelectasis, and preventing cyclic premature closure and opening of the alveoli; it lowers the inspiratory threshold in patients with an auto-PEEP, stabilizes the upper airways, especially, during sleep, and increases the pulmonary compliance through the increase in end-expiratory volume in selected patients, such as patients with obesity (22).

In contrast, BiPAP consists of the application of two different airway pressures: one pressure during the expiration (equivalent to the PEEP) and a supplementary pressure during the inspiration commonly referred to as pressure support (PS). The PEEP added to the PS is called the inspiratory positive airway pressure (IPAP), which is the total pressure delivered during the inspiration (21). BiPAP has the same physiological effect as CPAP through its PEEP setting. In addition, the PS helps to relieve the inspiratory muscles and increases alveolar ventilation (22). BiPAP is recommended as the first choice therapy in hypercapnic respiratory failure (1). In *de novo* AHRF, CPAP mainly helps oxygenation through the recruitment of closed alveoli and the prevention of atelectasis (23). The addition of a PS can reduce the work of breathing if sufficient pressure is added (24) and diminish the inspiratory effort of the patient (25). However, there are potential harms associated with using BiPAP in *de novo* AHRF. The main concern is a disproportionate increase of pulmonary pressures and volumes leading to overdistension and cyclic closure and opening of non-ventilated alveoli. This may result in volumetric and barometric lung injury (26). Indeed, patients with *de novo* AHRF can present a high ventilatory drive with high inspiratory volumes that could be worsened with PS (24, 27). These patients with a high tidal volume (VT) before the introduction of NIV or during NIV have a higher risk of NIV failure and progression to IMV (27). A balance between the need to decrease the respiratory workload and the risk of self-inflicted lung injury must be considered (28).

## Clinical evidence before the COVID-19 pandemic comparing HFNO to COT in AHRF

A systematic review published in 2020 compiled evidence on HFNO in AHRF before the COVID pandemic (29). This network meta-analysis included 25 trials and 3,804 participants and compared various non-invasive respiratory support strategies. Compared to COT, HFNO was associated with a significant reduction of the need for intubation [risk ratio (RR) 0.76; 95% confidence interval (CI) 0.55–0.99] and a non-significant reduction of 30-day mortality (RR 0.87; 95% CI 0.62–1.15). Similarly, Rochwerg et al. (30) identified nine studies (2,093 patients) comparing HFNO to COT in AHRF of any cause with similar estimates: a significant reduction in the need for intubation (RR of 0.85; 95% CI 0.74–0.99) but no statistically significant reduction in 30-day mortality (RR 0.94; 95% CI 0.67–1.31). The effects of HFNO on key clinical outcomes are summarized in Table 1.

**Table 1:** Pooled effect estimates of high-flow nasal oxygen compared to conventional oxygen therapy before the COVID pandemic.

**Outcome**	**Pooled estimates Rochwerg et al. (2)**	**Pooled estimates Ferreyro et al. (29)**	**Pooled estimates Oczkowski et al. (3)**	**Lay summary of the evidence comparing HFNO to COT**
Mortality	RR 0.94 (95% CI 0.67–1.31)	RR 0.87 (95% CI 0.62–1.15)	RR 0.97 (95% CI 0.83–1.13)	HFNO may have little or no influence on mortality rate (low certainty)^2^
Invasive Mechanical ventilation	RR 0.85 (95% CI 0.74–0.99)	RR 0.76 (95% CI 0.55–0.99)	RR 0.89 (95% CI 0.77–1.03)	HFNO may reduce the risk of invasive mechanical ventilation (low certainty)^3^
Escalation of therapy^1^	RR 0.71 (95% CI 0.51–0.98)	NA	RR 0.76 (95% CI 0.43–1.34)	HFNO may reduce the rate of therapy escalation (low certainty)^3^

COT, conventional oxygen therapy; CI, confidence interval; HFNO, high-flow nasal oxygen; NA, not assessed; RR, risk ratio. ^1^Escalation of therapy is defined by Rochwerg et al. as an increase in oxygen support therapy, either to HFNO (if in the control group), NIV or invasive mechanical ventilation and defined by Oczkowski et al. (3) as an escalation toward NIV only. ^2^Low certainty: due to very serious imprecision. ^3^Low certainty due to imprecision and risk of bias by lack of blinding and lack of predetermined IMV criteria in some studies.

The main RCTs comparing HFNO and COT, including more than 50 patients, and reporting clinically relevant outcomes are detailed in Supplementary Table S1 (31–38). Studies were performed in different settings including ICU (31, 32, 39, 40), emergency unit (35, 36, 38, 41), and acute care ward (37). While pneumonia was the main cause of AHRF, some trials also included patients with cardiogenic edema (34–36), COPD exacerbations (34, 36), and asthma exacerbations (42, 43). Furthermore, three trials focused on patients with immunosuppression from various etiologies (32, 33, 37).

Based on this initial body of evidence, the European Respiratory Society and the European Society of Intensive Care Medicine recommended HFNO over COT in AHRF (2, 3). The American College of Physicians (ACP) recently published a similar guideline (4). While pooling data very similar to the ERS guidelines (3), they concluded that the overall effect of HFNO over COT in AHRF remained uncertain (4). International guidelines regarding HFNO in AHRF are summarized in Table 2. These different interpretations appear to be mainly driven by the use of different methods for the pooled estimates. The ACP guidelines used a Peto odds ratio and Hartung–Knapp–Sidik–Jonkman random effects model, which probably led to an exaggerated weight of two negative studies of a smaller sample size (35, 44). These two studies account for < 1% of the events (3/418) but for 14.4% of the weight of the pooled estimate in the ACP guidelines. Moreover, these two studies differed in their designs; Spoeletini et al. evaluated HFNO during breaks of NIV among patients with hypercapnic respiratory failure, whereas Makdee et al. included patients with acute pulmonary edema, and the study was prognostically imbalanced with patients allocated to COT being more likely to benefit from NIV. For these reasons, the inclusion of these two studies in the meta-analysis and their overweighting appear debatable, hence the different conclusions of the ACP guideline.

**Table 2:** International recommendations regarding HFNO in acute hypoxemic respiratory failure.

**Society (year)**	**Recommendation**
European Society of intensive Care Medicine (ESICM) 2020 (2)	We recommend using HFNO compared to COT for patients with hypoxemic respiratory failure (strong recommendation, moderate certainty evidence).
European Respiratory society (ERS) 2022 (3)	The ERS task force suggests HFNO over COT in patients with acute hypoxemic respiratory failure (conditional recommendation, moderate certainty of evidence).
American College of Physicians 2021 (4)	Benefits of HFNO compared to COT in acute respiratory failure are not clear.

HFNO, high-flow nasal oxygen; COT, conventional oxygen therapy.

Overall, the evidence published before COVID-19 relies on several open-label RCTs, detailed in Supplementary Table S1, and supports the use of HFNO in AHRF to reduce intubation, yet with an uncertain impact on mortality (31–38). Certainty of the available evidence is limited by the imprecision in effect estimates (2, 3, 45) and the risk of bias of included studies, in particular, the absence of blinding due to the nature of the intervention. Moreover, the included populations are heterogeneous in terms of settings and patient characteristics (AHRF etiology, immunosuppression, the inclusion of COPD or cardiac failure, etc.). It remains therefore unclear if the HFNO benefit applies to the majority of included patients or more specifically to some yet unidentified subgroups.

## Clinical evidence before the COVID-19 pandemic comparing HFNO to NIV in AHRF

### NIV vs. COT in AHRF

As discussed above, HFNO probably allows a reduction in IMV in AHRF compared to COT. However, NIV is also frequently used in AHRF and has been reported to reduce mortality and intubation rate in patients with cardiogenic pulmonary edema (46–48). Similarly, BiPAP has been shown to reduce mortality and the need for IMV in patients with COPD exacerbation with acute hypercapnic respiratory failure and is recommended in this context (1, 49, 50). This leads to the questioning of the place of HFNO vs. NIV in *de novo* AHRF.

The role of NIV in the management of *de novo* AHRF remains controversial (1). The first historical RCT comparing BiPAP to early IMV reported a lower rate of serious complications and a shorter duration of stay in the ICU when using BiPAP (51). This study included mainly *de novo* AHRF but some patients with acute pulmonary edema were included (51). Subsequent RCTs comparing NIV to COT in *de novo* AHRF patients have shown inconsistent results varying from benefit to harm (31, 52–56). Most RCTs reporting positive results included a significant percentage of patients with either acute pulmonary edema or COPD exacerbation (52, 53). After the exclusion of patients with pulmonary edema or COPD exacerbation, a meta-analysis of the 2017 official ERS/ATS clinical practice guidelines on NIV concluded that NIV may reduce IMV (RR 0.75, 95% CI 0.63–0.89) in patients with *de novo* AHRF. The effect on mortality was unclear (RR 0.83, 95% CI 0.65–1.05) (1).

In 2015, Frat et al. published the three-arm FLORALI trial, in which 330 patients with *de novo* AHRF mainly due to pneumonia were randomized to continuous HFNO, BiPAP (alternating with HFNO), or COT in a multicenter, open-label controlled trial. The trial did not show any difference in IMV rates (primary outcome) across groups. However, there was a significant difference in favor of HFNO in 90-day mortality when compared to both COT and BiPAP with an HR of 2.01 (95% CI 1.01–3.99) and 2.50 (95% CI 1.31–4.78) respectively. There was also a lower rate of IMV in the HFNO group compared to COT and BiPAP in the subgroups of more severe patients (PaO_2_/FiO_2_ ≤ 200) with an HR of 2.07 (95% CI 1.09–3.94) and 2.57 (95% CI 1.37–4.84), respectively. Although these secondary results must be considered exploratory, this was considered a worrisome signal for the potential harms of NIV in this population. In this context, the 2017 ERS/ATS guidelines did not recommend the systematic use of NIV in *de novo* AHRF in patients without immunosuppression and recommended restricting its use to well-selected patients in a secured environment with prompt access to IMV (1).

For immunosuppressed patients, IMV is associated with a higher rate of complications. Smaller trials reported statistically significant results with a lower rate of IMV and mortality when comparing BiPAP with COT (57, 58). Later trials with a larger population did not confirm these results (39, 59). A pooled analysis of RCTs in immunosuppressed patients with *de novo* AHRF showed that NIV compared to COT was associated with a decrease in mortality (RR 0.68, 95% CI 0.53–0.88), a decrease in the need for IMV (RR 0.71, 95% CI 0.58–0.87), and a decrease in the rates of nosocomial pneumonia (RR 0.39, 95% CI 0.20–0.76) (1). In this context, the use of NIV over COT is recommended for immunosuppressed patients with a moderate degree of certainty in the 2017 ATS/ERS guidelines (1).

A network meta-analysis in 2020 reported that the use of NIV in AHRF was associated with a lower mortality and intubation rate than the use of COT (29). This was true for both helmet and facemask NIV (29). The potential benefits of using a helmet include reduced air leaks and an increased tolerance, leading to the potential delivery of a higher level of PEEP (60). However, this meta-analysis included patients with post-operative conditions and did not systematically exclude patients with acute pulmonary edema and COPD. Moreover, immunosuppressed patients and non-immunosuppressed patients were unfortunately combined in the analysis (29).

### HFNO vs. NIV in AHRF

Several RCTs compared HFNO with NIV in AHRF (31, 61–63). RCTs reporting on relevant clinical outcomes and including more than 50 patients are detailed in Supplementary Table S2. These trials differed in terms of setting, population, and interventions. Shebl et al. (62) compared HFNO with BiPAP in 70 patients with interstitial lung disease. Osman et al. (63) compared helmet CPAP against HFNO in 188 patients with acute cardiogenic pulmonary edema. Doshi et al. (61) compared HFNO and BiPAP in 204 patients with undifferentiated respiratory failure in the emergency department including some patients with hypercapnia.

The recent European guidelines addressed the comparison between HFNO and NIV in AHRF (3). They identified three studies reporting on mortality and five on intubation (8, 31, 61, 62). Compared to NIV, HFNO may be associated with a non-significant mortality reduction (RR 0.77, 95% CI 0.52–1.14) and reduced intubation rate (RR 0.84, 95% CI 0.61–1.16) (Table 3) (3). The ACP guidelines included only two RCTs comparing HFNO to NIV (31, 61) and reported a reduced risk of intubation (RR 0.71; 95% CI 0.53–0.95) and mortality (RR 0.44; 95% CI 0.24–0.79) among patients treated with HFNO compared to NIV.

**Table 3:** Pooled effect estimates of high-flow nasal oxygen compared to non-invasive ventilation before the COVID pandemic.

**Outcomes**	**Pooled estimate Oczkowski et al. (3)**	**Pooled estimate Baldomero et al. (4)**	**Lay summary of the evidence comparing HFNO to NIV**
Mortality	RR 0.77 (95% CI 0.52–1.14)	RR 0.44 (95% CI 0.24–0.79)	HFNO compared to NIV may reduce all-cause mortality (low certainty) ^1^.
Invasive mechanical ventilation	RR 0.84 (95% CI 0.61–1.16)	RR 0.71 (95% CI 0.53–0.95)	HFNO compared to NIV may reduce intubations (low certainty) ^1^.

CI, confidence interval; HFNO, high-flow nasal oxygen; NIV, non-invasive ventilation; RR, risk ratio. ^1^Low certainty due to imprecision and indirectness.

International recommendations regarding the comparison between HFNO and NIV in AHRF are summarized in Table 4. Certainty of the evidence is limited by imprecision (45), inconsistency, possibly due to variable AHRF etiologies, severity and settings, and indirectness due to the weight of the FLORALI trial, which could have suboptimal BiPAP settings (31, 65). Indeed, the low PEEP (mean 5 cmH_2_O) and the relatively low duration of the intervention (8 h) as well as the possible lack of humidification have been pointed out as limitations of this trial, which could underestimate the magnitude of the effect of NIV (3).

**Table 4:** Recommendations comparing high-flow nasal oxygen with non-invasive ventilation in acute hypoxemic respiratory failure.

**Society**	**Recommendation**
European Respiratory Society (ERS) (3)	The ERS task force suggests the use of HFNO over NIV in ARHF. (Conditional recommendation, very low certainty of evidence)
American College of Physicians (4)	Compared to NIV, HFNO may reduce intubation, all-cause mortality, and hospital-acquired pneumonia, and improve patient comfort in initial AHRF management.
Surviving Sepsis Campaign: International Guidelines for Management of Sepsis and Septic Shock (64)	For adults with sepsis-induced hypoxemic respiratory failure, we suggest the use of HFNO over NIV. Weak recommendation, low quality of evidence

AHRF, acute hypoxemic respiratory failure; HFNO, high-flow nasal oxygen; NIV, non-invasive ventilation.

The recently published FLORALI IM (i.e., immunosuppressed) trial was not included in these guidelines (3, 4, 66). This multicenter, open-label, randomized trial assigned 300 immunocompromised adult patients with *de novo* AHRF, to HFNO or BiPAP alternating with HFNO. Patients with hypercapnic respiratory failure and cardiogenic pulmonary edema were excluded. The median PEEP, PS, and duration of BiPAP were 7, 7 cmH_2_O, and 11 h, respectively. Despite higher PEEP and longer duration of BiPAP than the previous FLORALI trial, there was no effect on the mortality rate at day 28 (36% in the HFNO group and 35% in the BiPAP group, absolute difference 1.2% [95% CI −9.6−11.9]). None of the other secondary outcomes differed between groups except better comfort with HFNO than with NIV [−4 mm on the visual analogic scale (IQR −18–4) vs. 0 mm (−16–17); *p* = 0.040] and better oxygenation in the NIV group [PaO_2_/FiO_2_ 199 (SD 91) vs. 143 mm Hg (SD 76); *p* < 0.001] (66).

Based on the pre-COVID evidence, NIV did not seem to be associated with significant benefits compared to HFNO. Moreover, HFNO appears easier to provide and less resource-consuming than NIV. In this context, we think that it is reasonable to use HFNO over BiPAP in the first intention in most patients with AHRF (31, 66). Of importance, CPAP was poorly studied in AHRF before the occurrence of SARS-CoV-2 (55, 56).

## Clinical evidence comparing HFNO to COT and/or NIV in patients with COVID-19

Several RCTs have been conducted during the COVID-19 pandemic to compare different respiratory support strategies. These trials are summarized in Table 5 and detailed in Supplementary Table S3 (7–14).

**Table 5:** Randomized controlled trials comparing high-flow nasal oxygen, non-invasive ventilation, and conventional oxygen therapy in COVID-19-related acute hypoxemic respiratory failure.

**Study**	**Patient number (AHRF criteria)**	**Intervention**	**Control**	**Results of interest**	**Methodological evaluation**
Ospina-Tascòn 2021	220 (PaO_2_/FiO_2_ < 200)	HFNO	COT	HFNO significantly decreased the need for IMV (34.3 vs. 51% HR 0.62; 95% CI 0.39–0.96).	Low risk of bias (Open label but predetermined IMV criteria), Limited cross over
Perkins 2021	1237 (FiO_2_ > 40%)	HFNO	COT CPAP	Significantly lower composite of IMV rates and mortality at 30 days with CPAP (36.3%) vs. COT (44.4%) absolute difference −8% (95%CI −15 to −1%). No significant difference between HFNO and COT (44.3 vs. 45.1%).	High risk of bias (pragmatic design without predetermined IMV criteria and open-label design). 23.6% crossover rate from COT group (8.4% received CPAP, 7.6% received HFNO and 7.6% received both CPAP and HFNO)
Crimi 2022	364 (PaO_2_/FiO_2_ < 300)	HFNO	COT	Escalation of respiratory support (IMV, CPAP, BiPAP as chosen by the physician) not statistically different (RR 0.79) (95% CI 0.59 to 1.05)	High risk of bias (open label design with escalation of respiratory support including escalation to CPAP and BiPAP as the primary outcome) Trial underpowered.
Bouadma 2022	333 with AHRF (O_2_ flow ≥6 L/min)	HFNO	COT CPAP	No difference in 28-day IMV (COT 41.4% vs. CPAP 43% vs. HFNO 43.8%)	Low risk of bias (open-label design but predetermined IMV criteria). 29.4% were non-adherent to COT with 26.6% of patients crossed from the COT to HFNO group.
Frat 2022	711 patients (PaO_2_/FiO_2_ < 200)	HFNO	COT	No difference in 28-day mortality [10% (36/357) with HFNO and 11% (40/354) with COT, absolute difference, −1.2% (95% CI −5.8 to 3.4)]. Significant reduction of IMV in the HFNO group (45% with HFNO vs. 53% with COT)	Low risk of bias (open-label design but predetermined IMV criteria).
Nazir 2022	120 (supplemental oxygen)	HFNO	COT	Higher rate of escalation of respiratory support (IMV, CPAP, NIV) in the group COT (10% in the group HFNO vs. 43.3% in the group COT)	High risk of bias (open label design with escalation of respiratory support including escalation to CPAP and BiPAP as the primary outcome)
Grieco 2021	109 (PaO_2_/FiO_2_ ≤ 200)	HFNO	Helmet BiPAP	No difference in days free of respiratory support at 28 days (BiPAP 20 (IQR, 0–25) vs. HFNO 18 (IQR, 0–22)). Secondary outcome: IMV significantly lower in the BiPAP group than in the HFNO group (30 vs. 51%; difference, −21% [95% CI, −38 to −3%]).	Low risk of bias (open-label design but predetermined IMV criteria)
Nair 2021	109 (Oxygen requirement)	HFNO	BiPAP	Intubation rate similar between groups at 48 h (33% NIV vs. 20% HFNO, RR 0.60, 95% CI 0.31–1.15, *p* = 0.12). Secondary outcome: Intubation rate at day 7 significantly lower in the HFNO (27%) compared to NIV group (46%) (RR 0.59, 95% CI 0.35–0.99).	High risk of bias (open-label design and the co-intervention (prone positioning in HFNO but not in NIV).

AHRF, acute hypoxemic respiratory failure; BiPAP, bi-level positive airway pressure; CI, confidence interval; COT, conventional oxygen therapy; CPAP, continuous airway pressure; FiO_2_, fraction of inspired oxygen; IMV, invasive mechanical ventilation; IQR, interquartile range; PaO_2_, partial pressure of arterial oxygen; HFNO, high-flow nasal oxygen, NIV, non-invasive ventilation.

Ospina-Tascòn et al. (7) randomized 220 COVID-19 patients with AHRF (PaO_2_/FiO_2_ < 200 mmHg) to receive HFNO or COT. This open-label multicenter trial reported a reduced need for IMV in the HFNO group compared to COT (34.3% in the HFNO group vs. 51% in the COT group, HR 0.62; 95% CI 0.39–0.96). Only one patient crossed over from the COT to the HFNO group. This trial was considered at low risk of bias except for the open-label design, which the authors tried to mitigate using pre-defined intubation criteria (67).

Crimi et al. (11) randomized 364 patients with confirmed SARS-CoV-2 pneumonia with mild AHRF (SpO_2_ ≤ 92% or PaO_2_/FiO_2_ < 300 and the need for oxygen therapy) to receive HFNO or COT (1:1). The primary outcome was a need for escalation to advanced respiratory support (IMV, CPAP, and BiPAP). No statistical difference was observed (RR 0.79; 95% CI 0.59–1.05). This trial included mostly patients with less severe hypoxemia and was underpowered due to a lower number of events than expected.

Frat et al. (14) randomized 782 patients with AHRF to HFNO vs. COT. The trial was initiated before the COVID-19 pandemic and was redesigned during the COVID-19 pandemic. A total of 711 patients with AHRF (PaO_2_/FiO_2_ < 200) due to COVID-19 were included in the final analysis. No difference in 28-day mortality was observed [10% with HFNO vs. 11% with COT, absolute difference, −1.2% (95% CI −5.8–3.4)]. A significant reduction of IMV was observed in the HFNO group [45% with HFNO vs. 53% with COT, absolute difference −7.7% (95% CI; −14.9 to −0.4%)]. The low crossover rate is a strength of this trial; however, limited by a lack of power to show a difference in mortality because of lower-than-anticipated mortality rates.

Perkins et al. (9) randomized 1,237 patients with confirmed SARS-CoV-2 infection with AHRF (SpO_2_ < 94% or less despite receiving 40% FiO_2_) to receive HFNO, CPAP, or COT in a pragmatic, open-label, adaptative, and randomized trial. The incidence of the primary composite outcome (requirement of IMV or mortality at 30 days) was significantly lower with CPAP (36.3%) as compared to COT (44.4%) absolute difference −8% (95% CI −15 to −1%) but was not significantly different with HFNO when compared to COT [44.3 vs. 45.1%, absolute difference −1% (95% CI −8 to +6%)]. The difference between CPAP and COT was mainly driven by the requirement of IMV. The lack of benefit of HFNO might be explained by the high number of crossovers with a total of 23.6% of patients crossing from COT to other respiratory support (8.4% received CPAP, 7.6% received HFNO and 7.6% received both CPAP and HFNO), the early termination of the trial and the pragmatic nature of the trial with the lack of predetermined criteria to proceed to IMV. Indeed, while the pragmatic design increases the external validity of the trial, the absence of predefined intubation criteria may increase the risk of bias, especially in the context of the open-label design. Adverse events occurred more often in the CPAP group than in HFNO and COT groups (34.2, 20.6, and 13.9%, respectively). Common adverse events in the CPAP group included the following: interface/therapy intolerance (5.8%), pain (5.5%), cutaneous pressure sore (12.1%), claustrophobia (12.1%), oral dryness (6.6%), and hemodynamic instability (11.3%).

Bouadma et al. (10) randomized 333 patients with confirmed or highly suspected COVID-19-related AHRF to receive HFNO, CPAP, or COT. There were no differences in the incidence of IMV between COT (29.4%), CPAP (31.4%), or HFNO (32.6%). Important limitations of this trial included a high crossover rate (26.6% of patients in COT crossed to HFNO) and lack of power due to a two times lower-than-expected rate of events.

Grieco et al. (8) randomized 109 patients with confirmed SARS-CoV-2 infection and moderate to severe AHRF (PaO_2_/FiO_2_ ≤ 200) to receive either BiPAP with a helmet interface or HFNO. No significant difference in the number of days free of respiratory support within 28 days was observed. Interestingly, the rate of IMV (secondary outcome) was lower in the helmet group than in the HFNO (30 vs. 51%, *p* = 0.03). These findings are exploratory, as the primary outcome did not reach a significant effect.

Finally, two smaller trials summarized in Table 5 have been published. One reported a reduction in the rate of escalation toward mechanical ventilation (IMV or NIV) when HFNO was compared to COT and the other one did not find a significant difference in the rate of intubation at 48 h when HFNO was compared to BiPAP (12, 13).

Arabi et al. (15) randomized 320 patients with AHRF (PaO_2_/FiO_2_ < 200) related to COVID-19 to helmet NIV vs. usual respiratory support (facemask NIV, HFNO, COT, or a combination of these at the discretion of the physician). There was no difference in 28-day mortality (27% in the helmet group and 26.1% in the usual respiratory support group). There was no significant difference in any of the pre-specified secondary outcomes. The main strength includes the low crossover rate in both groups. The main limitations of this trial include the different controls (COT, HFNO, and Facemask NIV) and the lack of power (estimated mortality rates of 40%). More than 36.5% of the patients discontinued helmet NIV because of reported intolerance after a median of 20.5 h of use (IQR 3–48 h).

Overall, the evidence regarding HFNO in AHRF due to COVID-19 seems concordant with the pre-COVID-19 evidence, with a possible decrease in the risk of IMV compared to COT and an uncertain effect on mortality. However, important differences in inclusion criteria, AHRF severity, standardization of intubation criteria, and crossovers lead to some inconsistencies across available trials. Furthermore, different interventions were compared including COT, HFNO, CPAP, BiPAP, and the use of different interfaces and ventilator parameters, with no optimal strategy yet identified.

## Discussion

The COVID-19 pandemic led to an important amount of new evidence regarding the benefits of HFNO in AHRF with more than 3,000 patients included in recent RCTs. Most studies conducted during the COVID-19 period reported positive results in favor of HFNO compared to COT regarding the need for IMV with an uncertain effect on mortality. These recent pieces of evidence are consistent with the pre-COVID evidence regarding HFNO for AHRF of other etiologies. Various phenotypic patterns of COVID-19-related AHRF have been described, some characterized by preserved lung compliance and others with a more restrictive respiratory pattern (68–70). Therefore, it may be questioned if the conclusions of the COVID-19 evidence apply to other forms of AHRF. However, the observed variability in phenotypes among patients with COVID-19 probably also occurs in AHRF of other miscellaneous causes (71). Taken together, these recent RCTs increase the level of evidence regarding the potential benefit of HFNO in AHRF. However, important heterogeneity was present in terms of settings, AHRF severity (as assessed by the variability of the PaO_2_/FiO_2_ ratios), standardization of intubation criteria, and crossovers leading to some inconsistencies across available trials. In particular, the important rate of crossovers from COT to HFNO in several pragmatic trials may have led to underestimating the benefits of HFNO in these trials. Moreover, the open-label design of available RCTs due to the nature of the intervention, and the absence of strictly pre-defined IMV criteria in a significant proportion of these trials constitute a potential risk of bias and must be acknowledged as an important limitation of the available evidence.

The evidence regarding the comparison between HFNO and other non-invasive respiratory support strategies is still more equivocal. Although pre-COVID pieces of evidence suggested a possible superiority of HFNO over NIV, largely relying on the FLORALI trial, RCTs conducted during the COVID-19 pandemic reported inconsistent results; some studies suggesting a possible superiority of CPAP or BiPAP over HFNO (8, 9), other no difference (10), or even superiority of HFNO over BiPAP (13).

As previously discussed, AHRF encompasses a wide range of phenotypes, etiologies, severity, and contributing factors. HFNO, CPAP, and BiPAP have different physiological effects and individual patient characteristics probably mitigate the clinical effect of the different non-invasive respiratory supports. Gattinoni et al. (68) conceptually described two distinct radiological and physiological patterns of COVID-19 pneumonia: Type L pneumonia characterized by ground-glass densities on Ct-scan, preserved lung compliance, and low recruitability occurring in the early phase of the disease, and Type H pneumonia characterized by extensive non-aerated compartments on imaging, high elastance, and high recruitability. Based on this conceptual model, they suggested that respiratory support strategies should be adapted to these different patterns. Although this would be an interesting hypothesis to explore, these different patterns often co-exist in real-life patients and were therefore not reported in the available randomized data evaluating HFNO or NIV. Moreover, the observed clinical heterogeneity was present not only between RCTs but also within available RCTs, and their subgroup analyses were not systematically reported in particular regarding physiological aspects of lung injury and mechanics. In the recovery trial, no interaction was reported between HFNO effect and age, gender, ethnicity, time from onset to randomization, or body mass index, while patients requiring higher FiO_2_ (>60%) tended to benefit more from HFNO than patients requiring lower FiO_2_. In the study by Ospina-Tascon et al., younger patients (< 60 years) and patients with lower IL-6 levels (< 100 pg/ml) appeared to benefit more from HFNO than older patients or patients with higher levels of IL-6, while no interaction was detected between HFNO effect and the severity of hypoxemia. In a *post-hoc* analysis of the HENIVOT trial, patients with low PaCo_2_ (< 35 mmHg) or severe hypoxemia (PaO_2_/FiO_2_ × dyspnea visual analogical scale < 30) tended to benefit from helmet ventilation (72). Finally, Crimi et al. did not report any interaction between HFNO effect, time to randomization, age, co-morbidities, or severity of hypoxemia. In this context of inconsistent results across secondary analyses, it remains difficult based on the current evidence to identify a subgroup of patients likely to benefit specifically from HFNO or other non-invasive respiratory support, until more studies specifically designed to explore these subgroup hypotheses are reported.

Delayed intubation is a matter of concern as NIV failure in AHRF is frequent and associated with increased mortality (73). A correlation between the duration of respiratory distress (time with hypoxemia and RR >25/min) and reduced lung compliance (driving pressure >14 cmH_2_O) has been reported (74). However, the optimal timing for intubation remains uncertain (75). For these reasons, early recognition of patients failing to respond to non-invasive respiratory support is crucial, so that the use of HFNO and NIV should, to pour opinion, be restricted to settings such as intermediate/respiratory care units or intensive care units, where the response to treatment can be closely monitored and treatment strategies quickly adapted (76). The ROX index [(SpO_2_/FiO_2_)/respiratory rate] has been validated as a simple predictor of failure in patients with AHRF receiving HFNO and might be helpful to evaluate patient response, guiding therapy adaptation and to avoid potential risks associated with delayed intubation (77, 78). Given its ease of use, excellent tolerance, and the potential benefits compared to COT, HFNO could arguably be the first option for patients with AHRF and may constitute an adequate comparator to evaluate the benefits of alternative or additional strategies such as CPAP or BiPAP in future trials.

## Conclusion

The COVID-19 pandemic was associated with an unprecedented increase in hospital admissions for AHRF and was an opportunity to enrich the available evidence regarding non-invasive respiratory support strategies in this condition. HFNO is a type of well-tolerated respiratory support that is relatively easy to provide and probably reduces the need for IMV in patients with AHRF compared with COT. This may contribute to reducing IMV-associated complications and preserving ICU capacity. Several studies suggested possible additional benefits of NIV in COVID-19 patients. However, no optimal supportive respiratory strategy has yet been defined due to heterogeneity in interfaces, ventilator settings, AHRF definitions. and results. Given important phenotypic differences among patients with AHRF, the use of non-invasive respiratory supports should probably be restricted to monitored units and tailored to patient tolerance and response to therapy.

## Author contributions

LG, TA, and CM designed and wrote the manuscript. CE and AK revised the work and contributed to critical appraisal. All authors contributed to the article and approved the submitted version.

## Conflict of interest

The authors declare that the research was conducted in the absence of any commercial or financial relationships that could be construed as a potential conflict of interest.

## Publisher's note

All claims expressed in this article are solely those of the authors and do not necessarily represent those of their affiliated organizations, or those of the publisher, the editors and the reviewers. Any product that may be evaluated in this article, or claim that may be made by its manufacturer, is not guaranteed or endorsed by the publisher.
