# Supplementary material for: High-flow nasal oxygen in acute hypoxemic respiratory failure: A narrative review of the evidence before and after the COVID-19 pandemic
Source: Front Med (Lausanne). 2022 Nov 25;9:1068327. doi: 10.3389/fmed.2022.1068327 (PMC9732102; doi:10.3389/fmed.2022.1068327)
Supplement: Supplementary file 1 [file Data_Sheet_1.docx]

**Table S1 Main studies comparing HFNO to COT in AHRF Before COVID-19**

| **Study** | Patients | Intervention | Comparison | Primary and secondary outcomes | Results of interest | Methodological evaluation |
| --- | --- | --- | --- | --- | --- | --- |
| Frat 2015  FLORALI | 310 patients with de novo AHRF (PaO_2_/FiO_2_ ≤300)  Exclusion criteria: PaCO_2_ >45mmHg, exacerbation of asthma, cardiogenic pulmonary oedema, severe neutropenia, hemodynamic instability, use of vasopressors, GCS ≤12, contraindications to NIV, urgent need for IMV, do-not-intubate order.  Etiologies of AHRF: community acquired pneumonia (64%), hospital acquired pneumonia (12%), extra pulmonary sepsis (5%), aspiration or drowning (1.6%).  Immunosuppression in 26% of patients. | HFNO initial flow rate of 50L/min and FiO_2_ of 100% adjusted to maintain a SpO_2_ ≥92%. | COT using a nonrebreather face mask at a flow rate ≥10L/min to maintain a SpO_2_ level of ≥92%.  NIV group detailed in table S2 | Primary outcome: intubation at day 28  Secondary outcomes; mortality, number of ventilator free days, and duration of ICU stay. Dyspnea assessed with the use of a 5-point Likert scale, and comfort with the use of a 100-mm visual-analogue scale | Intubation rate: 38% (40 of 106) in the HFNO group vs 47% (44 of 94) in the COT group  Number of ventilator-free days at day 28 significantly higher in the HFNO vs COT group: 24±8 days, vs. 22±10 P = 0.02).  Risk of death at 90 days significantly higher with COT versus HFNO 2.01 (95% CI, 1.01 to 3.99).  In the subgroup of patients with a PaO_2_/FiO_2_ ≤200mmHg, the IMV rate was significantly lower in the HFNO group compared to COT with a HR of 2.07 (95% CI 1.09 to 3.94)  Increased degree of comfort, reduction of dyspnea and decreased respiratory rate when using HFNO as compared to COT | Low risk of bias (open-label but predefined IMV criteria).  26 patients from COT group (27.7%) and 14 from HFNO group (13.2%) crossed to NIV for rescue therapy. Among them 19/26 and 9/14 patients were subsequently intubated. All patients were analysed with ITT analyses.  Trial underpowered to show an effect for the intubation rate.  HFNO applied for at least 2 days |
| Azoulay 2018  HIGH trial | 776 immunocompromised ICU patients with AHRF  Exclusion criteria: imminent death; anatomical factors precluding the use of a nasal cannula; hypercapnia indicating NIV, isolated cardiogenic pulmonary oedema indicating NIV; surgery within the last 6 days  Etiology AHRF: pneumonia (n=320), invasive fungal infection (n=91), underlying involvement of haematological disease (n=80), other (n=265)  Immunosuppression: Cancer 79%, immunosuppressive drugs 34% | HFNO with 50L/min flow and 100% FiO_2_ then adjusted to maintain a SpO_2_ ≥95%. | COT to achieve a SpO_2_ ≥95%. | Primary outcome: 28-day mortality.  Secondary outcomes: intubation by day 28, PaO_2_:FIO_2_ ratio over the 3 days after intubation, BR, ICU and hospital lengths of stay, ICU-acquired infections, and patient comfort and dyspnea. | No difference in 28-day mortality (35.6% vs 36.1%; difference, −0.5% [95% CI, −7.3% to +6.3%]; HR, 0.98 [95% CI, 0.77 to 1.24]).  No difference in IMV rate (38.7% vs 43.8%; difference, −5.1% [95% CI, −12.3% to +2.0%]).  No significant difference in ICU LOS, ICU-acquired infections, hospital LOS, patient comfort and dyspnea scores. | Low risk of bias (open-label, but primary outcome mortality).  Open-label multicentre RCT.  (32 hospitals in France),  Crossover: 7.7% of patients from COT crossed to HFNO (failure of COT and do-not-intubate orders).  Analysis performed in ITT |
| Lemiale 2015 | 100 immunocompromised ICU patients with AHRF defined as need for oxygen >6 L/min to maintain a SpO_2_ >95% or symptoms of respiratory failure (BR>30/min, intercostal recession, labored breathing +/- dyspnea at rest)  Exclusion criteria: PaCO2>45mmHg, IMV before ICU admission, need for immediate NIV or IMV  Etiology of AHRF: sepsis (n=50, 50%), cardiogenic pulmonary oedema (n=7, 7%), lung involvement by the underlying disease ((n=16, 16%), non-infectious pulmonary disease (n=12, 12%)  Immunosuppression: cancer including haematological malignancies (84%). | HFNO 40– 50L/min FiO_2_ 100% adjusted to maintain a SpO_2_ ≥95%. | COT to maintain a SpO_2_ ≥95% using venturi mask. Initially FiO_2_ of 60% then adjusted to maintain SpO_2_ >95% | Primary outcome: Need for IMV or NIV during the 2-h oxygen therapy period.  Secondary endpoints comfort, dyspnea, and thirst, as assessed hourly using a 0–10 visual analogue scale. | During the 2-h study treatment period, 12 patients required IMV or NIV without significant difference between the two groups (15 % with HFNO and 8 % with the Venturi mask, P = 0.36).  None of the secondary endpoints differed significantly between the two groups. | High risk of bias due to the open-label design and the type of outcome (escalation towards NIV or IMV). Use of predetermined criteria for the need of IMV or NIV to try to mitigate that risk.  Multicentre (4 ICU in France), parallel group, randomized controlled trial.  No crossover allowed  Underpowered to detect a significant difference (calculation based on a 30% escalation to NIV or IMV in COT group and an absolute 20% difference) |
| Jones 2016  (HOT-ER study) | 303 adults patients with AHRF in the emergency department  Etiologies of AHRF: COPD exacerbation (n=79, 23%), pneumonia (n=72, 24%), cardiogenic pulmonary oedema (n=43, 14%), asthma (n=20, 7%), mixt (n=46, 15%) and other/unknown (n=44, 15%) | HFNO initial flow rate of 40L/min and FiO_2_ of 28% and titrated as indicated. | COT via a Hudson mask, Venturi device, or standard nasal cannula using wall oxygen titrated with a flow meter  (1–15 L/min) as indicated. | Primary outcome: escalation of treatment (NIV or IMV) in the emergency department using BTS guidelines.  Secondary outcomes: 90 day mortality, escalation of treatment within 24 h, In-hospital mortality, emergency department length of stay, hospital length of stay, time to escalation of treatment in emergency department, time to escalation of treatment in ward. | There was no difference in the primary outcome between the groups. In the HFNO vs COT group, 3.6% (95% CI 1.5–7.9%) vs 7.2% (95% CI 3.8–13%) required mechanical ventilation in the emergency department (P =0.16), and 5.5% (95% CI 2.8–10.2%) vs 11.6% (95% CI 7.2–18.1%) required mechanical ventilation within 24 h of admission (P =0.53).  There was no difference in mortality or length of stay.  Adverse effects were infrequent; one in 12 subjects did not tolerate HFNO. | High risk of bias (open-label design and the possible decision of the clinician to proceed to IMV/NIV based on personal decision)  Single centre, pragmatic, randomized controlled trial, New Zealand  Transition to NIV or IMV using the criteria of the BTS guidelines.  Underpowered (size of recruitment not met and fewer treatment escalation than predicted)  16 patients crossed from HFNO to COT. |
| Makdee 2017 | 128 adults patients with cardiogenic pulmonary oedema (history of acute dyspnea, bilateral rales on physical examination, and signs of pulmonary congestion on initial chest radiograph), a SpO_2_ <95% on room air, and a BR >24 breaths/min.  Exclusion criteria: need for immediate IMV or NIV, presence of myocardial infarction, GCS score <13, hemodynamic compromise, pregnancy, respiratory failure, end-stage renal disease, contraindications to the use of equipment with positive airway pressure, and concomitant pneumonia. | HFNO with flow rate of 35 L/min increased to 60 L/min if tolerated. FiO_2_ to maintain oxygen ≥95% | COT delivered by a nasal cannula or nonrebreather mask. | Primary outcome: BR 60 minutes post-intervention  Secondary outcomes: SpO_2_, pulse rate, blood pressure, severity of dyspnea (visual analogue scale ranging from 1 to 10), requirement for escalation to IMV or NIV within 24 hours, length of stay, mortality within 7 days, and pulmonary oedema grade as determined by chest radiograph findings | Baseline HFNO and COT mean BR were 28.7 breaths/min (SD, 3.2) and 28.6 breaths/min (SD, 3.5). Mean BR at 60 minutes post intervention were significantly lower in the HFNO group (21.8 versus 25.1 breaths/min; difference 3.3; 95% CI 1.9 to 4.6).  No significant differences in the admission rate, ED and hospital lengths of stay, NIV, IMV, or mortality. | High risk of bias because of the open-label design and the outcomes measurements (BR).  Monocentric (Thailand), randomized trial in the emergency department  Use of predefined criteria to escalate to NIV or IMV  Only patients with mild to moderate cardiogenic pulmonary oedema included (the other patients direct had NIV or IMV as indicated) |
| Bell 2015 | 100 patients with BR ≥25 breaths per minute and SpO_2_ ≤93% with shortness of breath  Exclusion criteria: Patients requiring immediate NIV or IMV (at the discretion of the physician), trauma patient, suspected pneumothorax, inability to provide consent (altered mental state, dementia, developmentally delayed, intoxicated, delirium).  Etiologies of AHRF: COPD exacerbations (n=45, 45%), respiratory infection (n=19, 19%), cardiogenic pulmonary oedema (n=22, 22%), others (n=8, 8%). | HFNO initial flow of 50 L/min and FiO_2_ of 30% titrated according to the patient condition. | COT using standard nasal prongs or facemask (Hudson, Venturi system or nonrebreathing mask). Titration according to the patient condition. | Primary outcome: need to escalate ventilation therapy (NIV; IMV, HFNO for patient on COT therapy) or a reduction in BR >20% within 2 h of commencement  Secondary outcomes: any reduction in the self-reported Borg score within 2 h of treatment, self-reported comfort score at 1 h post intervention, disposition from ED (admission to ward, ICU, or discharge from  ED), and length of stay in ED. | HFNO was associated with a higher proportion of patients who had >20% reduction in BR (66.7% vs 38.5%. P = 0.005). Two patients in the HFNO (escalated towards NIV) were escalated to more invasive therapy compared with 10 in the COT group (2 escalated to NIV, 1 escalated to IMV, 7 escalated to HFNO).  Significantly decreased dyspnoea scale, (75% from the HFNO group reported a reduction in Borg score compared with 55.8% from the COT group (P = 0.044).  No difference in other outcomes | High risk of bias (open-label design and the type of outcome measure (BR measurement), or the pragmatic design (escalation if treatment without objective criteria), crossover considered as an escalation in the COT group towards the HFNO group, No flow chart (information about patients screened and excluded missing).  Possible selection bias  Randomized trial in emergency department setting (2 centres in Australia).  Crossover from COT to HFNO is considered an escalation therapy (7/10 patients escalated towards HFNO in the arm COT). HFNO is also the tested intervention. There is the same number of patients escalated to NIV and IMV in both groups. |
| Apltekinoglu Mendil 2021 | 100 patients diagnosed with haematological malignancy and with AHRF (1: PaO_2_/FiO_2_ < 300mmHg or SpO_2_ < 92% on room air, 2: PaCO_2_ ≤ 45mmHg, 3: BR > 22 breaths/min or laboured breathing with respiratory distress)  Exclusion criteria: refusal to participate, pregnancy, need for immediate IMV or NIV, need for ICU admission, hemodynamic instability (mean arterial pressure <65mmHg), cardiogenic pulmonary oedema, patients unable to cooperate.  Etiology of AHRF: pneumonia (n=74, 74%), extra-pulmonary sepsis (n=10, 10%), others (n= 26, 26%) | HFNO initial flow rate of 30L/min and increased up to 50L depending on patient tolerability. FiO_2_ started at 100% FiO_2_ titrated for a SpO_2_ >94%. | COT via nasal prongs or venturi mask adjusted to keep a SpO_2_ of >94%. | Primary outcome: IMV at 7 days  Secondary outcome: mortality at 28 days, need for IMV and NIV at 28 days, visual analogue scale score for dyspnea and sensation of thirst first 7 days. | IMV needed in 10 patients in COT group (20%) and 17 patients in HFNO group (33%) (p = 0.14). Median visual analogue scale for comfort score at 2 and 24 hours were not different between HFNO and COT. 28-day mortality was 36.7% (18 deaths) in COT groups and 45% (23 deaths) in HFNO group (p=0.39)  Globally patients included had Mild hypoxemia (median PaO_2_/FiO_2_ = 257mmHg and 276mmHg in groups HFNO and COT respectively. | High risk of bias (open-label design, no clear predetermined criteria for IMV, absence of a clear analysis plan before the unblinding of the results, no power calculation). Use of predetermined criteria for treatment failure necessitating transfer to the ICU to mitigate the risk of bias.  Single centre, Turkey.  Normal ward of haematology. |
| Ko 2020 | 67 patients with cardiogenic pulmonary oedema.  Inclusion criteria: age >19 with a diagnosis of HF according to the NYHA classification I–IV within one year of admission; and acute pulmonary oedema confirmed by a chest radiograph at admission.  Exclusion criteria: non-cardiogenic pulmonary oedema; pneumonia; pregnancy; GCS <9; presence of a serious congenital heart condition; on-going dialysis; suspected myocardial infarction; poor chance of survival due to a pre-existing condition; O_2_ supply alone not being sufficient and the need for immediate IMV management; refusal to participate; inability to provide consent; do-not-resuscitate status. | HFNO initial flow rate of 45L/min and FiO_2_ of 100%. FiO_2_ and flow rate (up to 60 L/min) adjusted to maintain a SpO_2_ >93% | COT using nasal cannula or facemask at a flow rate >2 L/min. The flow rate was continuously adjusted to maintain a SpO_2_ of > 93%. | Primary outcome: changes in BR, parameters  of ABG, and lactate clearance in HF patients with acute pulmonary oedema.  Secondary outcomes: rate of IMV within 24h after ED admission, ICU admission rate, and all-cause mortality within 28 days of ED admission in each treatment group. | There were significant differences in favour of HFNO for the BR, SpO_2_ and PaO_2_ in the initial, 30 min, and 60 min measurements between the HFNO and COT groups (cf study for details)  There was no significant difference in secondary outcomes. | High risk of bias due to the absence of blinding and the type of outcome measure (BR measurement)  Randomized trial in the emergency department setting (two centres Korea).  In the study protocol, all patients had to undergo treatment with the assigned modality for at least 60 min. However, according to predetermined criteria of early termination, early intubation and escalation of other devices were allowed if the patients had an intolerable response to the sustained oxygen therapy with either the COT or HFNO. Early termination criteria included failure to tolerate the therapy as specified in the study protocol. |

AHRF: acute hypoxemic respiratory failure, BiPAP: bi-level positive airway pressure; BR: breathing rate; BTS: British Thoracic Society, CI: confidence interval; COT: conventional oxygen therapy; COPD: chronic obstructive pulmonary disease; CPAP: continuous airway pressure; ED: emergency department; GCS: Glasgow coma scale; FiO_2_ fraction of inspired oxygen; ICU: intensive care unit; IMV: invasive mechanical ventilation; IQR: interquartile range; PaO_2_: partial pressure of arterial oxygen; HFNO: High Flow Nasal Oxygen, NIV: non-invasive ventilation

**Table S2 Main studies comparing HFNO to NIV Before COVID-19**

|  | | | | | | |
| --- | --- | --- | --- | --- | --- | --- |
| Study | Patients | Intervention | Comparison | Primary and secondary outcomes | Results of interest | Methodological evaluation |
| Frat 2015  FLORALI | 310 patients with de novo AHRF (PaO_2_/FiO_2_ ≤300)  Exclusion criteria: PaCO2>45mmHg, exacerbation of asthma, cardiogenic pulmonary oedema, severe neutropenia, hemodynamic instability, use of vasopressors, GCS ≤12, contraindications to NIV, urgent need for IMV, do-not-intubate order.  Etiologies of AHRF: community acquired pneumonia (64%), hospital acquired pneumonia (12%), extra pulmonary sepsis (5%), aspiration or drowning (1.6%).  Immunosuppression in 26% of patients. | HFNO initial flow rate of 50L/min and FiO_2_ of 1.0 adjusted to maintain a SpO_2_ ≥92%. | BiPAP: through a facemask. PS adjusted to obtain an expired tidal volume of 7-10 ml/Kg, PEEP 2-10 cmH_2_O. FiO_2_ or PEEP adjusted to maintain a Spo2 ≥92%. Minimal NIV duration 8 hours per day for at least 2 days applied during sessions of at least 1 hour Between BiPAP sessions, HFNO, as described above  COT group described in table S1 | Primary outcome: intubation at day 28  Secondary outcomes; mortality, number of ventilator free days, and duration of ICU stay. Dyspnea assessed with the use of a 5-point Likert scale, and comfort with the use of a 100-mm visual-analogue scale | Intubation rate in the HFNO and NIV groups of 38% and 50% respectively (P = 0.18 for all comparisons).  Number of ventilator-free days at day 28 significantly higher in the high-flow–oxygen group compared to NIV groups (24±8 days, vs. 19±12; P = 0.02 for all comparisons).  Significantly higher risk of death at 90 days for BiPAP compared to HFNO with HR of 2.50 (95% CI 1.31 to 4.78)  In the subgroup of patients with PaO_2_/FiO_2_ ≤200mmHg, risk of IMV significant higher for patient in BiPAP group compared to HFNO with HR of 2.57 (95% CI 1.37 to 4.84)  No difference in the rate of complications between the three intervention | Low risk of bias (open label but predefined IMV criteria).  26 patients from COT group (27.7%) and 14 from HFNO group (13.2%) crossed to NIV for rescue therapy. Among them 19/26 and 9/14 patients were subsequently intubated. All patients were analysed with ITT analyses.  Trial underpowered to show an effect for the intubation rate.  HFNO applied for at least 2 days |
| Coudroy et al 2022  FLORALI-IM | 300 adults immunocompromised patients with AHRF, defined as BR ≥25 breaths/min and a PaO_2_/FiO_2_ ≤300mmHg.  Exclusion criteria: hypercapnia >50mmHg, patients who could strongly benefit from NIV (i.e. those with underlying chronic lung disease, with cardiogenic pulmonary oedema, or who were postoperative), severe shock, impaired consciousness defined as GCS ≤12, urgent need for IMV, do not intubate order, and contraindication to NIV.  Etiology of AHRF: infectious pneumonia (n=147, 49%), toxic (n=16, 5%), extrapulmonary sepsis (n=12, 4%), cardiogenic pulmonary oedema (n=12, 4%), haemoptysis (n=5, 2%), aspiration pneumonia (n=4, 1%), other (N=103, 34%)  Etiology of immunosuppression: Haematological malignancy (n=151, 50.3%), solid cancer (n=73, 24%), AIDS (n=12, 4%), organ transplant (n=35, 12%), corticosteroids or immunosuppressive therapy (n=190, 63%), leukopenia or neutropenia (n=44, 14%), stem cell transplantation (n=41, 14%), other (n=28, 9%). | HFNO continuously with a flow rate of 60 L/min or the highest tolerated.  FiO_2_ was adjusted to obtain an adequate SpO_2_ ≥92% | BiPAP alternating with HFNO.  BiPAP first session of at least 4 h, and then 12 h a day, with a dedicated ICU ventilator, targeting a tidal volume below 8 mL/kg of predicted bodyweight, and with a positive end-expiratory level of at least 8 cm H_2_O.  FiO_2_ was adjusted to obtain an adequate SpO_2_ ≥92%  HFNO was delivered as in the control group. | Primary outcome: mortality at day 28  Secondary outcomes: mortality in the ICU, in hospital, at day 90 and at day 180, intubation at day 28, length of stay in the ICU and in hospital, number of ventilator-free days at day 28, and tolerance. | No difference in mortality rate at day 28 (36% in the HFNO group vs 35% in the NIV group with absolute difference 1.2% (95% CI –9.6 to 11.90). None of the other prespecified secondary outcomes were different between groups except greater discomfort reduction with HFNO than with NIV (–4 mm on visual analogic scale [IQR –18 to 4] *vs* 0 mm [–16 to 17]; p=0.040) and better oxygenation with NIV than HFNO (PaO_2_/FiO_2_ 199 mmHg [SD 91] *vs* 143 mmHg [SD 76]; p<0.001). | Low risk of bias (open label but predefined intubation criteria)  Multicentre, open-label, randomized trial conducted in 29 ICUs (28 in France and one in Italy).  Median PEEP 7cmH2O and PS 7cmH2O with VT of 9.6mL/Kg (above the predefined protective ventilation target). Median duration of use of 11 hours. |
| Osman et al 2021 | 188 adults patients with tachypnoea (BR >30/min), desaturation (SpO2<90% while breathing oxygen >15L/min via a non-rebreather facemask) and dyspnoea due to cardiogenic pulmonary oedema.  Acute cardiogenic pulmonary oedema defined as the presence of the following clinical criteria: sudden onset of respiratory distress/respiratory failure, signs of respiratory fatigue, orthopnoea, bilateral rales on auscultation, pulmonary congestion on chest radiograph, and sonographic interstitial syndrome (multiple, bilateral, and homogeneously distributed B-lines at lung ultrasound), without medical history or signs suggesting pulmonary aspiration or infection.  Exclusion criteria: 1) altered mental status (GCS <8), 2) hemodynamic instability requiring vasopressors or inotropes/cardiogenic shock/cardiac arrest, 3) respiratory distress due to primary lung cause, 4) clinical need for urgent intubation, or 5) acute myocardial ischemia requiring urgent percutaneous coronary intervention 6) hypercarbia. | HFNO: FiO_2_ between 0.21 and 1.0, and gas flow up to 60 L/min through a heated humidifier (temperature 37°). FiO2 titrated for a SpO_2_ >94% and maintained throughout the study period. | Helmet CPAP with a gas flow set at a minimum of 40 L/min to prevent rebreathing. FiO_2_ was set up to 0.6 and PEEP was initially set at 5 cmH_2_O, then titrated upward with increments of 3–5  cmH_2_O (if needed), to achieve a SpO_2_ >94%. | Primary outcome : reduction in BR (measured by the investigator with a timer and by chest auscultation)  Secondary outcomes: included changes in heart rate, PaO_2_/FiO_2_ ratio, heart rate, acidosis, consciousness, oxygenation, and Respiratory rate (HACOR) score, Dyspnoea Scale, and intubation rate. Data were collected before CPAP/HFNO placement and after 1h of treatment. | CPAP was more effective than HFNO in reducing respiratory rate [-12 (95% CI; 11–13) vs. -9 (95% CI; 8–10), P < 0.001] and was associated with greater heart rate reduction [-20 (95% CI; 17–23) vs. -15 (95% CI; 12–18), P = 0.042], PaO2/FiO2 ratio improvement [-149 (95% CI; 135–163) vs. -120 (95% CI; 107–132), P = 0.003] as well as in HACOR scores [6 (0–12) vs. 4 (2–9), P < 0.001] and dyspnoea Scale [4 (1–7) vs. 3.5 (1–6), P = 0.003].  No differences in IMV rate were noted | High risk of bias Open label design and the type of outcome measure (BR measurement by the investigator)  Single centre (Malaysia) randomized controlled trial in the emergency setting.  Use of predefined failure criteria included  Median level of PEEP for helmet CPAP was 9cmH2O.  High targeted SpO_2_ >94%. |
| Doshi et al 2018 | 204 adults patients with undifferentiated respiratory failure with an indication to NIV based on clinical judgment  Exclusion criteria: suspected drug overdose, cardiovascular instability (hypotension requiring immediate intervention), end-stage cancer, life expectancy less than 6 months, significant respiratory depression on presentation (e.g., drug overdose), GCS <9, cardiac or respiratory arrest on presentation, need for emergency intubation, known or suspected cerebrovascular accident, known or suspected ST-segment elevation myocardial infarction, and patients with increased risk of pulmonary aspiration, agitation, or uncooperativeness.  Some patients with hypercapnic respiratory failure were included with a mean baseline PaCO_2_ level of 53.4 mmHg in the HFNO group and 58.7 mm Hg in the BiPAP group, and 60% of the patients enrolled (n=121) had a baseline PaCO_2_ of greater than 45 mmHg.  Etiology of acute respiratory failure: acute decompensated heart failure (n=44, 20.5%), acute COPD exacerbation (n=53, 30%), pneumonia (n= 53, 17%), asthma (n=7, 3%) | HFNO with a flow rate set to 35 L/min, with a starting temperature between 35°c and 37°c and FiO_2_ at 100% Adjustments in flow and temperature were made to alleviate respiratory distress and optimize comfort.  FiO_2_ was adjusted to maintain a SpO_2_ >88%. | BiPAP initiated with an oronasal mask, with inspiratory and expiratory positive airway pressures (IPAP, EPAP) set at the lower end of the following settings and increased as necessary to alleviate respiratory distress: IPAP 10 to 20 cmH_2_O (or 5 to 15 cm H_2_O above EPAP), and EPAP 5 to 10 cmH_2_O. FiO_2_ was initiated at 1.0. | Co-primary outcomes: 1) therapy failure at 72 hours defined as the need for intubation and 2) arm failure rate at 72 hours defined as the number of crossover to the alternate therapy and the number of patient with intubation. Noninferiority margins were set at 15% and 20% absolute difference percentage points, respectively.  Secondary outcomes: ability of HFNO versus NIV to affect the degree and timing of changes of PaCO2, pH, and other signs or symptoms of respiratory distress, including vital signs and perceived exertion scores reported by the patients | The intubation rate (HFNO =7%; BiPAP =13%; risk difference=–6%; 95% CI –14% to 2%) and any failure of the assigned arm (HFNO =26%; BiPAP=17%; risk difference 9%; confidence interval –2% to 20%) at 72 hours met noninferiority. The effect on PaCO_2_ over time was similar in the entire study population and in patients with baseline hypercapnia. Vital signs and blood gas analyses improved similarly over time. | High risk of bias (open label design and the possible use of clinical judgement alone to escalate therapy).  Multicentre (5 centres, USA), non-inferiority, randomized controlled trial in the emergency setting.  Mean IPAP 13cmH_2_O, mean EPAP 6cmH_2_O.  The effect on PaCO_2_ was similar in group HFNO and BiPAP even in the subgroup of patient with hypercapnia.  Crossover: group HFNO 23 patients crossed to the group BiPAP (22%) considered as “arm failure” outcome. 6 patients crossed from BiPAP group to HFNO group (5%).  Use of medium flow (35L/min) only |
| Shebl et al 2018 | 70 adults patients with ILD and AHRF (PaO2/FiO2 ≤300mmHg) despite oxygen supplementation at a flow rate >10 L/min for at least 15 min or if they had manifestation of increased work of breathing, for example, use of accessory muscles of respiration  Exclusion: Presence of pneumothorax, absolute indication for urgent IMV or contraindications to NIV.  Etiologies of ILD: idiopathic pulmonary fibrosis (IPF) (n= 19, 27%), hypersensitivity pneumonitis (n=9, 13%), connective tissue disease (n=9, 13%), drug-induced ILD (n=2, 3%), Langerhans cell histiocytosis (n=4, 6%), pneumoconiosis (n=3, 4%), sarcoidosis (n=7, 10%), non-IPF-idiopathic interstitial pneumonia (n=17, 10%).  Etiology of AHRF: pneumonia (n= 39, 55.7%), acute exacerbation of fibrosis (n=21, 30%), pulmonary thromboembolism (n=5, 7.1%) patients and cardiac failure (n=5, 7.1%). | HFNO with FiO_2_ from 0.21 to 1.0, and a flow up to 60 l/min. | BiPAP with PEEP gradually incremented to 12 cmH_2_O. PS was added if respiratory acidosis (pH<7.35) or if BR >30 breaths/min, and FiO_2_ was adjusted at the lowest level to maintain a PaO_2_ >60 mmHg | Primary outcome: IMV rate within 28 days  Secondary outcomes: in hospital mortality and ventilator free days within 28 days | No difference in IMV rate (20.6% in the HFNO group and 22.2% in the NIV group (P=0.87)).  The ventilator-free days at day 28 was significantly higher in the HFNO group (20±5 vs. 16±7 days in the NIV group; P=0.008). The rate of in-hospital mortality was not different for HFNO compared to NIV (26.5% vs 30.6% respectively (P=0.71)). | High risk of bias due to unclear concealment, no calculation of power/alpha error (possible type one error on the number of days without ventilation), absence of blinding and some subjective predetermined criteria for IMV  Single centre (Saudi Arabia) randomized trial.  BiPAP is not commonly recommended for ILD patients with AHRF and the comparison of HFNO to BiPAP is questionable  No cross over. |

AHRF: acute hypoxemic respiratory failure, AIDS: acquired immunodeficiency syndrome; BiPAP: bi-level positive airway pressure; BR: breathing rate; CI: confidence interval; COT: conventional oxygen therapy; COPD: chronic obstructive pulmonary disease; CPAP: continuous airway pressure; ED: emergency department; GCS: Glasgow coma scale; FiO_2_ fraction of inspired oxygen; ICU: intensive care unit; IMV: invasive mechanical ventilation; IQR: interquartile range; PaO_2_: partial pressure of arterial oxygen; HFNO: High Flow Nasal Oxygen, NIV: non-invasive ventilation

**Table S3: Studies comparing HFNO with COT or NIV in AHRF caused by COVID-19.**

| Study | Patients | Intervention | Comparison | Primary and secondary outcomes | Results of interest | Methodological evaluation |
| --- | --- | --- | --- | --- | --- | --- |
| Ospina-Tascòn et al 2021  (HiFLo-Covid) | 220 adults with COVID-19, respiratory distress and a ratio PaO_2_/FiO_2_ <200mmHg.  Inclusion: suspicion or confirmation of SARS-CoV-2 infection (PCR test), PaO_2_/FiO_2_ <200mmHg accompanied with respiratory distress signs (e.g. use of accessory muscles, and BR >25/min), and <6 hours elapsed since fulfilling the criteria of acute respiratory failure  Exclusion criteria: need for immediate IMV, PaCO_2_ >55mmHg, pregnancy, high suspicion of cardiogenic pulmonary oedema; history of left ventricular ejection fraction <45%, history of chronic heart failure, clinical suspicion or confirmation of demyelinating disease, history of advance COPD patients or hospitalisation due to COPD decompensation within the last year, advanced liver cirrhosis, conditions precluding the use of a HFNO, do-not-intubate or do not resuscitate orders; imminent death. | HFNO: continuously applied through large-bore nasal prongs using heated and humidified gas at an initial flow of 60 L/min and an FIO2 of 100%. FiO_2_ adjusted to maintain a SpO_2_ ≥92%. Flow rate decreased in patients reporting discomfort due to high-flow oxygen therapy. HFNO therapy continuously applied. | COT oxygen applied continuously through any low-flow oxygen device or combination thereof (nasal prongs, mask with or without oxygen reservoir,  Venturi mask systems). Rates of gas flow and FiO_2_ adjusted to maintain SpO_2_ ≥92% until patient intubation or recovery. | Primary outcome: IMV rate  Co-primary time to clinical recovery until day 28 as assessed by a 7-category ordinal scale.  Secondary outcomes: proportion of patients requiring early IMV, IMV–free days within 28 days, kidney replacement therapy–free days, hospital and ICU lengths of stay, overall mortality by day 28, proportion of serious adverse events, and proportion of bacterial and fungal infections. | HFNO significantly decreased the need of IMV (34.3% in HFNO group vs 51% in COT group, HR 0.62, 95% CI 0.39-0.96).  No difference in the median time to clinical recovery within 28 days was 11 days in HFNO group vs 14 days in COT group (-3.0 95% CI -7.5 to 1.0) and clinical recovery within 28 d of 77% in HFNO group vs 71% in COT group (HR 1.39, 95% CI 1.00-1.92).  Secondary outcomes:  HFNO was related to lower risk of intubation at days 7 and 14 (HR 0.59 (95% CI 0.38-0.94) and 0.63 (95% CI 0.41-0.97) respectively). median ventilator-free days within the first 28 days after randomization was higher in HFNO group compared to COT (28 vs 24 respectively adjusted odds ratio, 0.77; P = .01).  Other secondary outcomes were not statistically different. | Low risk of bias (open label, but predefined IMV criteria)  Randomized trial in 3 hospitals in Colombia (the emergency department and intensive care unit).  Randomization 220 patients, 199 analysed (8 patients withdrew consent, 13 patients transferred to other hospitals within 72H and were excluded from the analysis). In this context intention-to-treat analysis not strictly followed.  Only 1 patient crossed over (COT to HFNO).  Prone position was allowed in both groups |
| Crimi et al 2022 | 364 patients with confirmed COVID-19 (PCR test and pathological imaging), SpO_2_ ≤92% or PaO_2_/FiO_2_ <300mmHg and the need for oxygen therapy.  Exclusion criteria: BR ≥28/min, severe dyspnea and/or use of accessory muscles, PaO_2_/FiO_2_ ≤200mmHg, need for immediate IMV or NIV according to clinical judgment, septic shock, evidence of MOF, GCS<13, neuromuscular disease, PaCO_2_ >45mmHg, patient with home oxygen or home CPAP/BiPAP and limitation of care. | HFNO up to 60L/min according to patient tolerance (mean= 51L/min), temperature from 37 to 31°C according to patient comfort (mean temperature 32°) | COT with a Venturi mask preferred but any other devices possible. FiO_2_ titrated to SpO_2_ between 92 and 96%. | Primary: Escalation of respiratory support (IMV, CPAP, NIV as chosen by the physician).  Secondary outcomes: rate of clinical recovery, time to the escalation of respiratory support, type of respiratory support as the first-line escalation therapy by day 28, admission to ICU, hospital and ICU length of stay, dyspnoea score (range, 0 (no dyspnoea) to 10 (severe dyspnoea)), patient comfort score (range, 0 (severe discomfort) to 10 (perfect comfort)), SpO_2_/FiO_2_ ratio divided by BR (ROX index), National Early Warning Score 2, mortality at 28 and 60 days, and in-hospital, days free from CPAP/NIV/IMV, oxygen free days, treatment intolerance. No blinding of adjudication was performed for outcome assessments. | No difference in escalation of respiratory support (absolute difference: -8.2% (95% CI -18 to +0.14%). Primary escalation to CPAP (68 patients), NIV (46 patients) and IMV (11 patients).  There was no difference in secondary outcomes except for a statistically significant improvement in median dyspnoea score on the first time point at 2 hours and on days 3, 4 and 5 was found in the HFNO group compared to COT. | High risk of bias (open label design and the measure of escalation therapy outcome with some subjective criteria to escalate)  Randomized, parallel-group, multi-centric trial (27 centres, in Italy, Spain, Greece, Turkey, Poland, Portugal  Mild hypoxemia (PaO_2_/FiO_2_) in between 200 and 300mmHg)  Power calculation not reached (80% power for an absolute difference of 20% based on an escalation in group COT of 55%). Observed escalation in group COT of 38.6%.  Predetermined escalation criteria: PaO_2_/FiO_2_ ≤180mmHg with FiO_2_ ≥50% and at least one of the following: BR≥28/min, severe dyspnea, signs of increased work of breathing e.g. use of accessory muscles |
| Frat et al 2022  (SOHO-COVID tiral) | 711 adult patients with COVID-19 related AHRF (PaO_2_/FiO_2_ <200mmHg)  Inclusion criteria: suspected or confirmed diagnosis of COVID-19 (PCR test), pulmonary infiltrate and PaO_2_/FiO_2_ <200 mmHg while breathing oxygen at a flow rate >10L/min for at least 15 minutes.  Main exclusion criteria: PaCO_2_ >45mmHg, exacerbation of COPD, cardiogenic pulmonary edema, hemodynamic instability defined by signs of hypoperfusion or use of vasopressors (>0.3 μg/Kg/min), GCS <13, urgent need of IMV, do-not intubate order, refusal to participate. | HFNO continuously delivered via large bore binasal prongs with gas flow of 50L/min or more and heated humidified air. The FiO_2_ was adjusted to target a SpO_2_ between 92 and 96%. HFNO applied for at least 48 hours and stopped and switched to COT when the patient maintained a SpO_2_ of at least 92% and a BR <25/min with a FiO_2_ <41% | COT continuously delivered through a nonrebreathing mask with oxygen flow set at 10 L/min or more adjusted for a SpO_2_ between 92 and 96% until recovery or IMV | Primary outcome: mortality at 28 days.  Secondary outcomes: the proportion of patients who required IMV within 28 days, number of ventilator-free days at day 28, mortality at various predefined times (in the ICU, in the hospital, and up until day 90), length of stay in the ICU and hospital, level of oxygenation at 1 hour, dyspnea level, comfort level, intervals between randomization and intubation, intervals between intubation criteria and intubation procedure, Sepsis-related Organ Failure Assessment score during the first 48 hours after intubation and the overall incidence of serious adverse events | There was no difference in the mortality rate when HFNO was compared to COT at day 28 (10% for HFNO vs 11% for COT, absolute difference of -1.2%, 95% CI -5.8 to 3.4%).  IMV rate was significantly lower in HFNO group than COT (45% vs 53%, absolute difference -7.7%, 95% CI -14.9 to -0.4%).  Other secondary outcomes showed no significant difference. | Low risk of bias (open label and mortality for the primary outcome measure and use of predetermined criteria for IMV)  Lack of power (estimated mortality rate of 25% for power calculation).  Low crossover rate  Randomized controlled trial, open-label, multicentric, in 34 ICUS in France. |
| Perkins et al 2021  RECOVERY-RS) | 1237 patients with confirmed SARS-CoV-2 pneumonia with AHRF randomized to receive HFNO (418 patients), CPAP (380 patients) or COT (475 patients). Randomization 1:1:1 or 1:1 if one of the oxygenation support devices unavailable.  Inclusion: SpO_2_ ≤94% despite receiving 40% FiO_2_ and deemed suitable for IMV if indicated.  Exclusion: patient with immediate need for IMV, known pregnancy.  Based on the judgment of the clinician in charge, the presence of a contraindication to the intervention precluded randomization to that specific trial group. | HFNO titrated according to local hospital policy. Heated and humidified with mean flow at initiation of 52.4L  or  CPAP: FiO_2_ and PEEP titrated according to local hospital policy. Mean PEEP of 8.3cmH_2_O. Continuous use except for breaks accorded for comfort | COT standard facemask or nasal cannula | Primary outcome: composite of IMV or mortality within 30 days.  Secondary outcomes: individual incidence of IMV or mortality within 30 days, time to IMV, duration of IMV, time to death, mortality (ICU or hospital stay), admission to the ICU, length of stay in the ICU, and length of stay in the hospital (included time from emergency department arrival to hospital discharge). | Requirement of tracheal intubation or mortality within 30 days was significantly lower with CPAP (36.3%) vs COT (44.4%) absolute difference -8% (95% CI -15% to -1%) but was not significantly different with HFNO (44.3%) vs COT.  There was a significant difference in the individual incidence of tracheal intubation within 30 days in the CPAP group (33.4% vs 41.3% in the COT group; absolute difference, −8% [95% CI, −15% to −1%]) but no statistical difference for mortality alone.  Significantly fewer participants in the CPAP group required admission to the ICU compared to the COT group (55.4% vs 62.9%, respectively; absolute difference, −7% [95% CI, −15% to −3%]). Among the participants who required IMV, there was a statistically significant increase in the median time to tracheal intubation in the CPAP group (2.0 days [IQR, 1.0 to 4.0 days]) compared with the COT group (1.0 day [IQR, 0 to 4.0 days]) (median difference, 1.0 day [95% CI, 0.2 to 1.8 days]).  For all other outcomes and comparisons, there was no statistically significant difference between study groups. | High risk of bias (open label and pragmatic design with no specified criteria for IMV). At time of IMV, same physiological characteristics.  Randomized, parallel group, open-label, adaptative, 3 groups, trial. Multicentric (48 acute care hospitals) in UK and Jersey.  Difference in primary outcome for CPAP versus COT mainly driven by the rate of IMV  Part of the treatment (CPAP, HFNO, COT) performed out of the ICU (medical wards)  High crossover: 17.1% of participants (15.3% in the CPAP group, 11.5% in HFNO group and 23.6% in the COT group).  Adverse events occurred in 34.2% of participants in the CPAP group, 20.6% in the HFNO group and 13.9% in the COT group and was statistically different. Common adverse events frequently encountered in the CPAP group were: interface/therapy intolerance (5.8%), pain (5.5%), cutaneous pressure sore (12.1%), claustrophobia (12.1%), oral dryness (6.6%) and haemodynamic instability (11.3%). Serious adverse events were more frequent in the CPAP group (1.8%) than HFNO (0%) or COT (0.2%) p = 0.002. There were 8 serious adverse events in the CPAP group including one death. Serious adverse events were judged probably, possibly, unlikely or unrelated to CPAP use in 1, 3, 2 and 1 patients respectively.  Study is underpowered (planned recruitment 3000 patients for a reduction of 5% in the primary outcome) and early termination of trial due to the pandemic waves and recruitment issue |
| Bouadma et al 2022  (COVIDICUS) | 546 adult patients with confirmed or highly suspected COVID-19 related AHRF randomized for high versus standard dose of dexamethasone and then to a non-invasive respiratory support (HFNO, CPAP and COT 1:1:1). 213 patients not eligible for randomization of oxygenation support (92 needing immediate IMV, 101 with contraindication to one or more oxygenation support strategies). 333 patients randomized for oxygen support intervention (109 COT, 109 CPAP, 115 HFNO).  Inclusion: admission to ICU within 48 hours, confirmed or highly suspected COVID-19, AHRF (defined as PaO_2_ <70mmHg, SpO_2_ <90%, BR >30/min, signs of laboured breathing, respiratory distress or need for O_2_ flow ≥6L/min)  Exclusion criteria: IMV at inclusion, anatomical factors precluding use of nasal cannula, PaCO_2_ >50mmHg, intolerance at admission to any of the oxygenation strategies, limitation in life-sustaining treatment, active untreated bacterial, fungal, or parasitic infection and hypersensitivity to dexamethasone. | HNFO: humidification with flow of 30L/min titrated and adjusted based on the clinical response up to 60L/min. FiO2 titrated for the targeted SpO_2_.  CPAP: started at 15-30 L/min oxygen (which corresponds to an average pressure of 4-10 cmH_2_O). The level was decreased or increased as needed based on the clinical response and tolerance. For at least the first 6 to 12 hours, CPAP was given continuously and then discontinuously (for at least 6 hours/day) based on patient tolerance. | COT using non- rebreather facemask until IMV, death or fulfilment of oxygen delivery cessation criteria (SpO_2_ >92% without oxygen and BR <25/min) | Primary outcome measurement for oxygenation intervention: fulfilment of criteria for IMV assessed at 28 days.  Secondary outcomes: healthcare–associated infection at day 28, number of IMV-free days alive at day 28, ICU and hospital lengths of stay (LOS) and the 28-day cumulative incidence of actual IMV. | No difference in 28-day cumulative incidence of IMV criteria fulfilment (41.4% for COT, 43% for CPAP, and 43.8% for HNFO with HR 1.08 (95% CI 0.71 to 1.63) for CPAP vs COT and 1.04 (95% CI 0.69 to 1.55) for HFNO vs COT)  Overall, none of the interventions elicited any significant differences in secondary outcomes vs standard of care. | Low risk of bias (open-label design concerning the oxygenation support device but the use of predetermined IMV criteria fulfilment as primary outcome)  Randomized controlled trial, multicentric in 19 ICU units, in France.  Important crossover rate (e.g. 26.6% of COT patients crossed to HFNO)  Power calculation not reached (planning IMV of 80%, but only 41.4% reached in the study)  58 patients filled IMV criteria but were finally not intubated (cumulative incidence of actual IMV at day 28: 28.6%, 31.4% and 32.6% for COT, CPAP and HFNO respectively) . |
| Nazir et al 2022 | 120 patients with COVID-19 related AHRF  Inclusion criteria: dyspnea with BR between 24-30/min, SpO_2_ 90-94% on room air, fever and cough.  Exclusion criteria: severe COVID-19 pneumonia, GCS ≤12, patients with primary pulmonary disease, tracheostomy, or any nasal/facial defect that could impede HFNO. | HFNO flow rate of 40 - 60 L/min, FiO_2_ of 80-100% adjusted to a SpO_2_ ≥96-99% | COT using a non-rebreathing facemask used at a flow rate of 12 - 15 L/min and FiO_2_ between 80-100%, adjusted to maintain SpO_2_ ≥96-99% | Primary outcome: progression-free survival without escalation of an oxygen delivery device.  Secondary outcomes: change of PaO_2_, PaO_2_/FiO_2_, BR, heart rate, mean arterial pressure, number of patients requiring NIV, number of patients requiring IMV, time for de-escalation of oxygen therapy, the time to progression to severe disease, survival at day 28, and patient satisfaction level. | Significantly higher number of patients successfully treated in HFNO group without escalation of oxygen delivery device in the group HFNO compared to COT (90% compared to 56.6%, *p*<0.001).  HFNO significantly decreased the time for de-escalation of oxygen therapy and the BR and increased oxygenation (cf study). No significant difference in the other outcomes | High risk of bias (open-label design and the outcome of escalation towards a NIV or IMV). The authors used predetermined criteria to mitigate the risk of bias (cf study)  Randomized controlled trial in a single ICU centre in India.  No cross-over of patients |
| Grieco et al 2021  (HENIVOT) | 109 patients with COVID-19 related AHRF (PaO_2_/FiO_2_ ≤ 200mmHg).  Exclusion criteria: PaCO_2_ >45mmHg, history of chronic respiratory failure or moderate to severe cardiac insufficiency, acute exacerbation of chronic pulmonary disease and kidney failure. | Helmet BiPAP (PEEP 10-12cmH_2_O; PS 10-12cmH_2_O) continuously for at least 48 hours eventually followed by HFNO | HFNO with gas flow set at 60 L/min and eventually decreased in case of intolerance.  FiO_2_ titrated to obtain p SpO_2_ between 92% and 98%. Humidification set at 37° or 34° according to the patient’s comfort | Primary outcome: Number of days free of respiratory support within 28 days  Secondary outcomes included the proportion of patients who required IMV within 28 days, the number of days free of IMV at day 28, the number of days free of IMV at day 60, in ICU mortality, in hospital mortality, 28-day mortality, 60-day mortality, ICU length of stay and hospital length of stay. | No difference in the median days free of respiratory support within 28 days after randomization were found: 20 (IQR, 0-25) in the helmet group and 18 (IQR, 0-22) in the HFNO group (mean difference of 2 days, 95% CI −2 to 6).  7 of the 9 prespecified secondary outcomes showed no significant difference. The rate of IMV was significantly lower in the helmet group than in the HFNO group (30% vs 51%; difference, −21% [95% CI, −38%to −3%]).  The median number of days free of invasive mechanical ventilation within 28 days was significantly higher in the helmet group than in the HFNO group (28 [IQR, 13-28] vs 25 [IQR 4-28]; mean difference, 3 days [95%CI, 0-7]). | Low risk of bias (open-label design but prespecified criteria for IMV)  Multicentric in 4 ICUs in Italy. |
| Nair et al 2021 | 109 adult patients with COVID-19 related AHRF (BR > 30/min and/or SpO_2_ <90% breathing room air).  Exclusion criteria: hemodynamic instability and requirement of high dose vasopressor therapy, pregnancy, COPD/chronic respiratory failure, morbid obesity, patients with urgent requirement of IMV, severe hypoxia (SpO2 < 90% with BR >40/min for >10/min), severe hemodynamic impairment with altered mentation, GCS < 8, or cardiac arrest. | HFNO initially set at 50L/min and FiO_2_ at 100%. The flow and FiO_2_ were subsequently adjusted between 30-60L/min and 50-100%, respectively to maintain SpO2 >94%. | BiPAP applied with either a mask or helmet device with a pressure support of 10-20 cmH_2_O adjusted with the aim of obtaining an expired tidal volume of 7-10mL/Kg of predicted body weight and PEEP of 5-10 cmH_2_O and FiO_2_ between 50 and 100% titrated to target a SpO_2_ >94%. | Primary outcome: Intubation by 48 hours.  Secondary outcomes: improvement in oxygenation by 48 hours, IMV rate at day 7, and in-hospital mortality. | IMV rate was similar between the groups (33% NIV vs 20% HFNO, RR 0.6, 95% CI 0.31-1.15).  Intubation rate at day 7 was lower in the HFNO (27%) compared to NIV group (46%) (RR 0.59, 95% CI 0.35-0.99) and this difference remained significant after multiple adjustments. Other outcome did not show any significant difference between the 2 groups. | High risk of bias (open-label design and the co-intervention (prone positioning in HFNO but not in NIV). Risk of bias mitigated but the use of predefined IMV criteria  Randomized controlled trial in a single centre (India) |

AHRF: acute hypoxemic respiratory failure, BiPAP: bi-level positive airway pressure; BR: breathing rate; CI: confidence interval; COT: conventional oxygen therapy; COPD: chronic obstructive pulmonary disease; CPAP: continuous airway pressure; ED: emergency department; GCS: Glasgow coma scale; FiO_2_ fraction of inspired oxygen; ICU: intensive care unit; IMV: invasive mechanical ventilation; IQR: interquartile range; PaO_2_: partial pressure of arterial oxygen; PEEP: positive end-expiratory pressure; PS: pressure support; HFNO: High Flow Nasal Oxygen, NIV: non-invasive ventilation; MOF: Multi organ failure
